# Research on the Node Importance of a Weighted Network Based on the *K*-Order Propagation Number Algorithm

**DOI:** 10.3390/e22030364

**Published:** 2020-03-22

**Authors:** Pingchuan Tang, Chuancheng Song, Weiwei Ding, Junkai Ma, Jun Dong, Liya Huang

**Affiliations:** 1 College of Electronic and Optical Engineering & College of Microelectronics, Nanjing University of Posts and Telecommunications, Nanjing 210023, China; 1018020705@njupt.edu.cn (P.T.); weiweiding001@gmail.com (W.D.); 1218022825@njupt.edu.cn (J.M.); 2 Bell Honors School, Nanjing University of Posts and Telecommunications, Nanjing 210023, China; B17090816@njupt.edu.cn; 3 Institute of Intelligent Machines, Hefei Institute of Physical Science Chinese Academy of Sciences, Hefei 230031, China; 4 National and Local Joint Engineering Laboratory of RF Integration and Micro-Assembly Technology, Nanjing 210003, China

**Keywords:** complex network, node importance, K-order propagation number, disease propagation

## Abstract

To describe both the global and local characteristics of a network more comprehensively, we propose the weighted *K*-order propagation number (WKPN) algorithm to extract the disease propagation based on the network topology to evaluate the node importance. Each node is set as the source of infection, and the total number of infected nodes is defined as the *K*-order propagation number after experiencing the propagation time *K*. The simulation of the symmetric network with bridge nodes indicated that the WKPN algorithm was more effective for evaluation of the algorithm features. A deliberate attack strategy, which indicated an attack on the network according to the node importance from high to low, was employed to evaluate the WKPN algorithm in real networks. Compared with the other methods tested, the results demonstrate the applicability and advancement that a lower number of nodes, with a higher importance calculated by the *K*-order propagation number algorithm, has to achieve full damage to the network structure.

## 1. Introduction

A complex network [1] is the abstract expression of a real system [2,3], where the nodes rely on edges to connect with each other. Node importance, which generally varies from node to node, is an important basis for designing the network structure, improving the system robustness, etc. [4,5,6,7]. When it comes to the analysis of node importance, most of the available methods focus on unweighted networks, in which only a single edge is allowed between any two nodes [8]. Nevertheless, weighted networks also have great applicability [9,10], as they are more similar to the networks abstracted from the real world, such as the transportation networks between cities, collaboration networks of scientists, etc.

Currently, several evaluation approaches of node importance for weighted networks have been proposed, primarily from two perspectives—local characteristics and global characteristics. For the former aspect, the weighted degree centrality, H-degree centrality, and weighted page-rank approaches are gradually becoming mainstream. For the latter, the weighted betweenness centrality method is the most commonly used.

The local characteristics of a node [11] reflect the properties of other nodes directly connected to it. The primary evaluation approach used to determine it is degree centrality [12]. In 2010, Opsahl et al. improved this approach and proposed the weighted degree centrality (WDC) [13] algorithm, which introduced the concept of strength, stating that the node would be more important if it gains greater strength in the network. However, the definition of the strength index is not unique, which may result in conceptual confusion.

For instance, the strength index of the nodes can be determined by the sum of the edge weights, the sum of flows [14], the number of the nodes’ neighbors with a weighted edge [15], and the amount of mutual information [16]. Due to the inconsistencies in concepts, the WDC algorithm is not universal. In addition, different from other WDC approaches, from the perspective of mathematical concepts, the WDC algorithm based on mutual information (MI) evaluates the node’s importance from the probability and statistics. Although the MI algorithm [16] expresses the interaction strength among nodes in weighted networks, directly connected nodes are also the only requirement to be considered.

Zhao S.X. et al. [17] put forward the H-degree centrality (HDC) algorithm extended from the Lobby index proposed by Korn [18] in 2011. If there are, at most, *n* edges connected to a node and the weight of the edges is not less than *n*, then the H-degree centrality of this node is *n*. H-degree centrality can be seen as a compromise between using the node strength and degree to measure centrality. However, there are several shortcomings in this method leading to low efficiency, e.g., the value of the edge weight is supposed to be in appropriate range or the node importance will not be sorted effectively. Hence, in various improvements based on degree centrality, local information can be reflected integrally but global information cannot.

In addition to the algorithm series of degree centrality, another algorithm focusing on local characteristics is the weighted page-rank (WPR) algorithm [19] proposed by Page et al. in 1999. The WPR algorithm was a method originally used by Google to identify the hierarchy of webpages. If Webpage A points to Webpage B through a hyperlink, this is equivalent to Webpage A voting for Webpage B. In addition, Page A is supposed to assign a part of its own Page-Rank value to Page B. Finally, just as the importance of a paper can be measured by the number of references cited in other papers, the importance of the webpage is judged according to the Page-Rank value.

As the degree of the bridge node in a network is likely to be small, the WPR algorithm may easily bring about underestimation of the node importance of the bridge node. Therefore, as the algorithms mentioned above focus greatly on the nodes’ local characteristics, it is difficult for them to evaluate special nodes, such as bridge nodes, which is a disadvantage of these algorithms.

Evaluation approaches reflecting the nodes’ global characteristics [20] make up for the aforementioned shortcomings, and the weighted betweenness centrality (WBC) algorithm [21] is a typical representative of them. The betweenness centrality [22] is the ratio of the number of edges passing through a node in the shortest paths to the number of all shortest paths in the network. In a weighted network, the path length between the nodes is determined by the edge weight. In the process of infectious disease propagation, the probability for closely-connected persons to become infected is larger, thus the reciprocal of the edge weight is often used to measure the distance. For example, if the weight of one edge is twice the other one, then the distance of the former is half of the latter.

Based on these principles, the weighted betweenness centrality (WBC) algorithm [21] is applicable to the world wide web (WWW) [23]. Nonetheless, the delay of all these networks and the interference among nodes are not sensitive to the nodes’s number [24,25,26]. In view of ignoring the nodes’ number mentioned above, it is hard to characterize the non-negligible effect of the nodes’ number on the transmission efficiency of the network. Therefore, when considering from only a global aspect, there is the possibility of overwhelming the local characteristics, which is disadvantageous in representing the node’s importance.

Hence, it is difficult to balance the local and global aspects for existing mainstream algorithms when evaluating the node importance. In this paper, we posit that the local and global characteristics should be described not only by directly-connected nodes but by all nodes in the network. Additionally, while both the local and global aspects should be considered, their influences are diverse in the various network structures. Therefore, as a combination of these two aspects, a new node importance evaluation approach “the weighted *K*-order propagation number (WKPN) algorithm” is proposed in this paper, in which *K* stands for the propagation stride.

As a comprehensive evaluation index, $c^{K}$, is defined to adjust the combined contribution of both sides in evaluating the node importance. When *K* is smaller, $c^{K}$ is likely to represent the influence of the local characteristics; when *K* is larger, $c^{K}$ tends to represent the global influence. The *K* value can vary from 0 to the network diameter *d*, which is exactly the process considering local and global characteristics comprehensively. In summary, the WKPN algorithm has good universality for different networks and significant effects on preserving both global and local characteristics as much as possible.

In this paper, a detailed description of the creation of the WKPN algorithm and its experiments on various networks is provided. The organization of this paper is as follows. In Section 2, we describe the establishment process of the WKPN algorithm in detail. In Section 3, we present experiments on both simulation networks and real networks and discuss the results. In Section 4, we give the conclusions obtained and the prospects for further research.

## 2. Weighted *K*-Order Propagation Number Algorithm

Models such as susceptible infective (SI), susceptible infective susceptible (SIS), and susceptible infective removed (SIR) [27] are widely used in information propagation, which were originally applied to the domain of disease transmission. Among them, whether individuals can be cured and have immunity are important factors giving rise to differences of the above models. In the SI and SIS models, they assume that individuals do not have immunity and the population is divided into susceptible and infected individuals. In addition, the SIS model presumes that infected individuals have a certain possibility to return to a susceptible state and may be re-infected, which is opposite to the SI model. Based on this, the SIR model adds a new category, called “the immune”, in addition to the two original types. However, in these three models, the disease propagation process is assumed to be random contact, with the topological relationship between individuals ignored.

Inspired by all the above models, we propose the WKPN algorithm by abstracting the simplest disease propagation process where the infected individuals cannot be cured in the complex network.

First, assume an undirected network graph G(V,E,W), in which $V=\{v_{1},v_{2},\cdots ,v_{n}\}$ is defined as the nodes set, $E=\{e_{ij}|0\leq i\leq n,0\leq j\leq n\}$ is the edges set, and $W=\{w_{ij}|0\leq i\leq n,0\leq j\leq n\}$ is the edge weights set. Among them, with the weight of $w_{ij}$, $e_{ij}$ represents the edge between the nodes $v_{i}$ and $v_{j}$.

Generally, there are two definition forms for edge weights: similar and dissimilar weight. For similar weight, a higher value corresponds to a shorter distance between the two nodes and vice versa. In this paper, similar weight forms are employed legitimately since the edge weight is defined as the disease propagation time. The smaller the edge weight is, the shorter the propagation time is, and the greater the node correlation is. Therefore, the similar weights are more available.

We assume that susceptible individuals can only be infected by direct contact with infected individuals. Then, the node $v_{i}$ and aggregate $\Gamma (v_{i})$ are defined as an infected source and the adjacent susceptible individuals. With respect to the node $v_{j}\in \Gamma \left(v_{i}\right)$, $v_{i}$ will spread disease to $v_{j}$ with $p_{ij}=1$, spending time $t_{ij}$ affected by the edge weight $w_{ij}$. In addition, if any node is affected by multiple infected sources in the propagation process, this method comprehensively evaluates that node.

By summarizing the above description of factors, such as the propagation probability and time consumption, the following hypotheses can be made:

**Hypothesis** **1.** *The infected individuals can only spread disease to those who are susceptible and adjacent.*

**Hypothesis** **2.** *The time consumption caused by the disease propagation process is the edge weight between the nodes.*

**Hypothesis** **3.** *A susceptible node will be transformed into an infected one once it is infected by any of its adjacent nodes.*

When considering the importance of a node, a common method is to measure the time required for that node to infect all nodes in the network. The less time is spent, the higher importance the node has. For a connected network, the total number of nodes propagated from any infected source in the network after a long time will be the same. To cope with this problem, the propagation time *K* is introduced as another significant parameter. The smaller *K* is, the more likely to represent local network features, while a larger *K* is more likely to be a global feature. In particular, K=0 indicates that the propagation process has not yet started.

According to **Hypotheses 1 and 3**, we can find the number of infected nodes $N_{v_{i}}^{K}$ after the propagation time of *K*, when setting $v_{i}$ as the source of infection:

(1) $$N_{v_{i}}^{K}=\sum_{v_{j}\in V}I\left(D\left(v_{i},v_{j}\right)\leq K\right),$$

where $N_{v_{i}}^{K}$ is named the *K*-order propagation number, in which $D\left(v_{i},v_{j}\right)$ represents the weight sum of the total edges through the shortest path from $v_{i}$ to $v_{j}$, and *I* is the indicator function. The larger $N_{v_{i}}^{K}$ is, the more important the node is in the scale of *K*. Equation (1) is an improved version of the weighted network from our former research [28] in unweighted networks. Moreover, when *K* is larger than *d*, which is the diameter of the largest connected part of a network, the $N_{v_{i}}^{K}$ of any nodes will not change with *K*. Therefore, the value of *K* can only fall between 0 and *d*.

It is clear that the value of the propagation time of *K* is the key to the evaluation of node importance. After that, according to $N_{v_{i}}^{K}$, the *K*-order structure entropy $H^{K}$ is defined based on the information entropy. In this way, the network heterogeneity can be evaluated [28] as:

(2) $$H^{K}=-\sum_{i=1}^{n}\frac{N_{v_{i}}^{K}}{\sum_{j=1}^{n}N_{v_{j}}^{K}}\log\left(\frac{N_{v_{i}}^{K}}{\sum_{j=1}^{n}N_{v_{j}}^{K}}\right),\;K\in \left[0,d\right].$$

The smaller the *K*-order structure entropy $H^{K}$ is, the stronger the heterogeneity of the networks is [28]. Former research [28] examined the heterogeneity of networks such as small world (WS) and scale-free (Barabasi-Albert (BA)) network. From the perspective of propagation process, the larger the value of $H^{K}$ is, the smaller the difference among various *K*-order propagation number $\left\{N_{{}^{v_{1}}}^{K},N_{{}^{v_{2}}}^{K},\cdots ,N_{{}^{v_{n}}}^{K}\right\}$ is, which is to set each node $\left\{v_{1},v_{2},\cdots ,v_{n}\right\}$ as the source of infection. The *K*-order structure entropy $H^{K}$ needs to consider various cases of *K* values, as both the local and global perspective of the impact on node importance are required. In summary, a comprehensive evaluation from K=0 to K=d is considered and the node importance $Q_{v_{i}}$ of node $v_{i}$ is defined as:

(3) $$\begin{array}{cc} \; & Q_{v_{i}}=\sum_{K=0}^{d}c^{K}\cdot S_{v_{i}}^{K},\\ \; & S_{v_{i}}^{K}=\frac{N_{v_{i}}^{K}-\min\left(N^{K}\right)}{\max\left(N^{K}\right)-\min\left(N^{K}\right)},N^{K}=\left\{N_{{}^{v_{1}}}^{K},N_{{}^{v_{2}}}^{K},\cdots ,N_{{}^{v_{n}}}^{K}\right\},\\ \; & c^{K}=1-\frac{H^{K}-\min\left(H\right)}{\max(H)-\min(H)},H=\left\{H^{0},H^{1},\cdots ,H^{d}\right\}, \end{array}$$

where $S_{v_{i}}^{K}$ is the normalized result of $N_{v_{i}}^{K}$ in order to avoid larger $N_{v_{i}}^{K}$ masking the smaller ones since $N_{v_{i}}^{K}$ usually grows with the increase of *K* dramatically. Therefore, this paper maps $N_{v_{i}}^{K}$ onto [0, 1], considering only the relative order of node importance. With respect to the weight coefficient $c^{K}$, we consider that the smaller the *K*-order structure entropy $H^{K}$ is, the larger the weight coefficient $c^{K}$ is. Equation (3) pays more attention to the moment when the difference of the node importance is relatively large and ignores the moment when the difference is small.

To summarize, $Q=\{Q_{v_{1}},Q_{v_{2}},\cdots ,Q_{v_{n}}\}$ is the aggregate of node importance calculated via the weighted *K*-order propagation number algorithm.

## 3. Node Importance Analysis for the WKPN Algorithm Based on a Deliberate Attack Strategy

To measure the features of the WKPN algorithm in the node importance assessment, comparisons were implemented, including a symmetric network with bridge nodes, the Science Museum visitor network [29], the Facebook forum network [30], the non-US airport routing network [31], and the US 500 busiest commercial airports network [32].

The deliberate attack strategy was employed to examine the node importance [33,34,35], which refers to attacking the corresponding node, that is, removing all the connecting edges of the node. In this way, the algorithms were evaluated by the characteristics of a complex network change with the attack. As isolated nodes may appear after the network is attacked, the network efficiency *e* was selected to evaluate the connectivity of the network. The expression of the network efficiency *e* is

(4) $$e=\frac{1}{n(n-1)}\sum_{v_{i}\neq v_{j}}\frac{1}{d_{v_{i}v_{j}}},$$

where $d_{v_{i}v_{j}}$ is the shortest path length between the nodes $v_{i}$ and $v_{j}$, and with the increase of the *e* value, the network efficiency is higher; when the network is totally composed of isolated nodes, *e* takes the minimum value of 0.

Attacks may give rise to an interruption of the network connection path; the shortest path between the nodes will increase and the network efficiency will decrease accordingly. To reflect the reduction of the network efficiency after the attack more intuitively, the network efficiency decline rate ε is defined as follows, according to former research [36],

(5) $$\varepsilon =1-\frac{e}{e_{0}},$$

where $e_{0}$ is the original network efficiency without an attack. ε increases as the attack progresses from 0 to 1. ε=0 when the network has not been attacked and ε=1 when all edges have been deleted.

### 3.1. A Symmetric Network with Bridge Nodes

**Figure 1:**
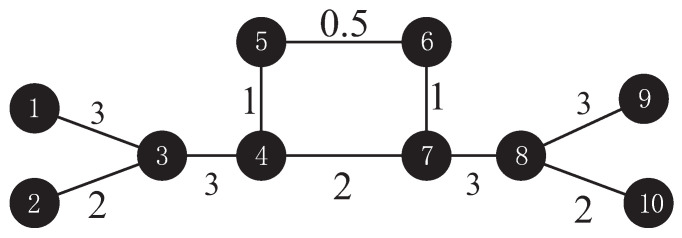
The weighted network topology with a symmetric network with bridge nodes.

First, a symmetric network with bridge nodes was taken as an example (as shown in Figure 1). The node importance aggregate *Q* was calculated via the WKPN algorithm to compare with the MI algorithm [16]. Table 1 is the node importance ranking, which was obtained by the above two algorithms.

There were some differences in evaluating node importance between the MI and WKPN algorithms. In the MI algorithm, the node importance of $v_{3}$ and $v_{8}$ was higher than $v_{4}$ and $v_{7}$, but the algorithm proposed in this paper gave the opposite conclusion. We adopted the deliberate attack to measure the node importance of these nodes. Table 2 gives the average efficiency value change of the network after deleting the corresponding nodes.

It is clear to see the decline of the average network efficiency after deleting any nodes, which indicates that the deletion weakens the information flow of the network to a certain extent. Nonetheless, it is difficult to neglect that the decline rate of the deleting nodes $v_{4}$ and $v_{7}$ is more than twice that of the deleting nodes $v_{3}$ and $v_{8}$. Thus, we consider that the node importance of $v_{4}$ and $v_{7}$ is higher than that of $v_{3}$ and $v_{8}$.

From the perspective of Figure 1, the nodes $v_{4}$ and $v_{7}$ are in the position with the largest global information control capability, which is equivalent to two “bridge nodes”. With the greatest degree and total edge weight, the network will no longer be connected if these two nodes are deleted. Thus, $v_{4}$ and $v_{7}$ are of the greatest importance. However, the degrees of $v_{3}$ and $v_{8}$ are less than those of $v_{4}$ and $v_{7}$. Hence, it is reasonable that the node importance of $v_{3}$ and $v_{8}$ ranked in second place.

Other sorting results in the WKPN algorithm were also consistent with the information shown in Figure 1. $v_{1}$, $v_{9}$, $v_{2}$, and $v_{10}$ were all connected to $v_{3}$ and $v_{8}$, which had exactly the same node importance; however, the total edge weight of former two nodes was higher than the latter two. Thus, $v_{2}$ and $v_{10}$ were ranked after $v_{1}$ and $v_{9}$. $v_{5}$ and $v_{6}$ were both at the margin of the network, which was intended to suffer less structural damage if they were deleted. Although both nodes were directly connected to the most important nodes, $v_{4}$ and $v_{7}$, the edge weight between them was tiny. Hence, $v_{5}$ and $v_{6}$ were considered to be of the least importance.

Therefore, the WKPN algorithm was more accurate in evaluating the node importance.

### 3.2. Real Networks

**Figure 2:**
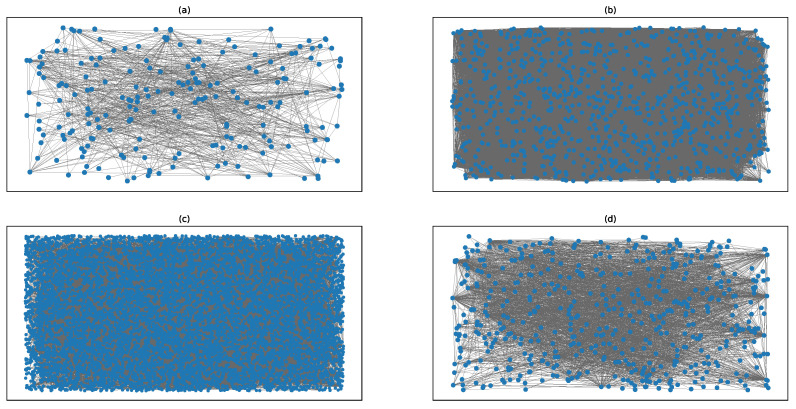
The networks graph structure: (**a**) the Science Museum visitor network; (**b**) the Facebook forum network; (**c**) the non-US airport route network; and (**d**) the US 500 busiest commercial airports network.

**Figure 3:**
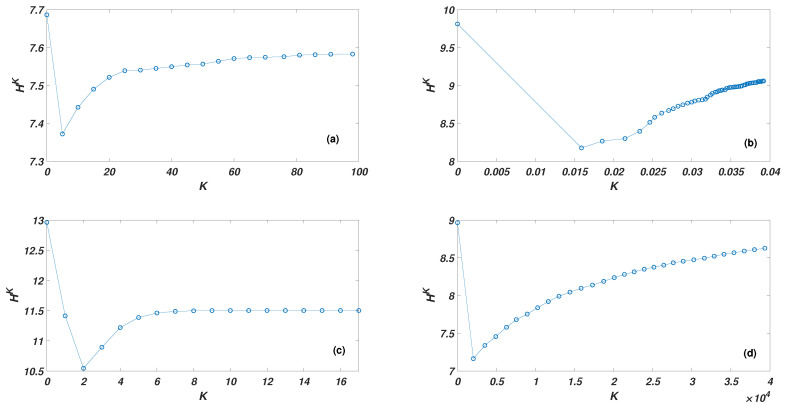
*K*-order structure entropy $H^{K}$ varies with K: (**a**) the Science Museum visitor network; (**b**) the Facebook forum network; (**c**) the non-US airport route network; and (**d**) the US 500 busiest commercial airports network.

To further verify the superiority of the WKPN algorithm, node importance research was conducted on certain real networks: the Science Museum visitor network, the Facebook forum network, the non-US airport routing network, and the US 500 busiest commercial airports network. The basic network features are shown in Table 3. The network graph structures are shown in Figure 2 and the *K*-order structure entropies are shown in Figure 3.

Due to the large number of nodes, in this section, the deliberate attack strategy refers to attacking the network concerning node importance from high to low. Considering the bias of node importance sorting before and after a deliberate attack, we updated the sorting result after every attack. In addition, if there were multiple nodes with equal node importance, the one with the minimum No. was selected to attack.

**Figure 4:**
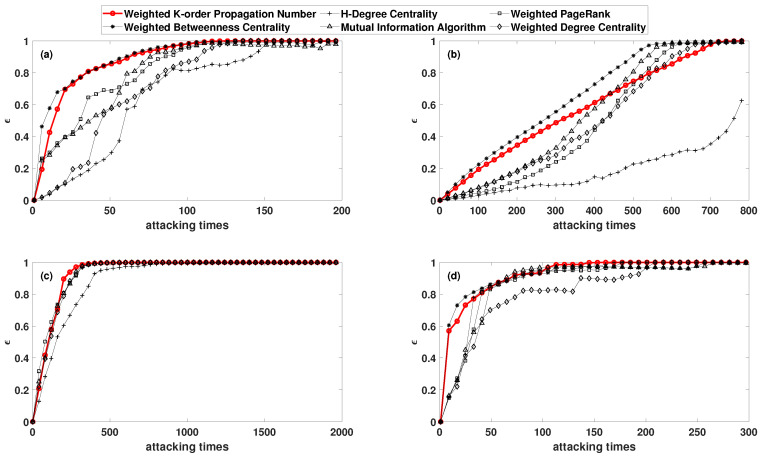
The network efficiency decline rate varies with the attack times: (**a**) the Science Museum visitor network; (**b**) the Facebook forum network; (**c**) the non-US airport route network; and (**d**) the US 500 busiest commercial airports network.

**Figure 5:**
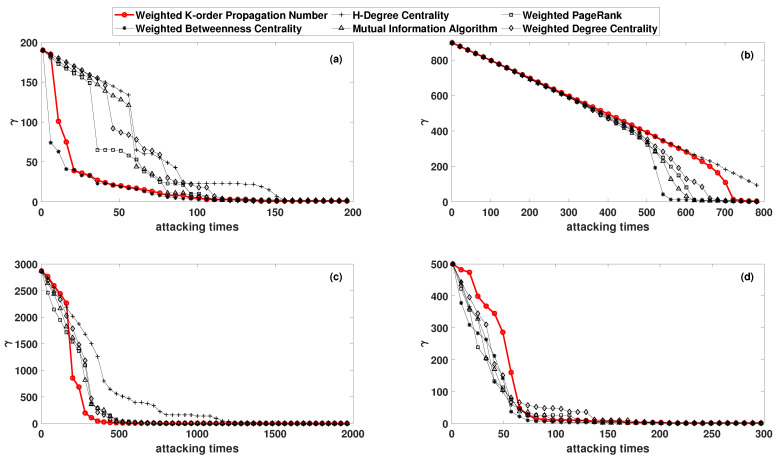
The node number of maximum sub-graph γ in the network varies with the number of attacks: (**a**) the Science Museum visitor network; (**b**) the Facebook forum network; (**c**) the non-US airport route network; and (**d**) the US 500 busiest commercial airports network.

Furthermore, to analyze the changes in the network topology before and after the attack, the node number of maximum sub-graphs in the network was set as γ according to former research [35]. The WKPN algorithm was applied to these four complex networks mentioned above, and the simulation comparison results (curves of ε and γ with attacking times) were obtained, as shown in Figure 4 and Figure 5. In particular, the damping coefficient of the Page-Rank is 0.5.

As for the Science Museum, the network efficiency declined the most rapidly when deliberate attacks were carried out according to the rank of the WKPN algorithm and the WBC algorithm. After approximately 70 attacks, the network efficiency dropped by nearly 90%. The MI algorithm and WPR algorithm required approximately 100 times, while the WDC algorithm required 120 times, and the HDC algorithm needed 150 times to achieve a similar effect. In addition, when the WKPN and WBC algorithm were employed to attack the network, the decline rate of the γ was much higher than the other methods.

We could also attack the network to a paralysis and compare the number of attack times. Taking the WKPN and WBC algorithm as examples, when the network was attacked 80 times, the node number of the maximum sub-graph γ was only 8, which is only 4% of the original network. The network was essentially paralyzed. To achieve the same paralysis, the MI algorithm, the WPR algorithm, and the WDC algorithm required 120 times, while the HDC algorithm needed more than 160 times.

For the Facebook forum network, the damage degree and damage trend of the network were relatively close after the deliberate attacks via the WKPN and WBC algorithm. The network efficiency declined more quickly in the early stage and more moderately in the later stage. For the non-US airport routing networks, the WKPN algorithm gave rise to the fastest decline rate of γ, the node number of the maximum sub-graph. For the US 500 busiest commercial airports network, the WDC algorithm had the worst attack capability, which was relatively close to the other algorithms. Although γ decreased slower in the early stage compared with other algorithms, it also paralyzed the network in a small number of times.

In summary, for each network mentioned above, deliberate attacks based on the WKPN algorithm needed to remove fewer nodes with a higher node importance to achieve full damage to the network structure.

## 4. Conclusions

In this paper, considering the correlation among individuals, we propose the weighted *K*-order propagation number algorithm, which is based on the improvement of the topological network structure in the infectious disease model. By simulating the symmetric network with bridge nodes, we found that the WKPN algorithm performed better in finding nodes with the “bridge” effect. We conducted simulation comparisons based on the deliberate attack strategy with the Science Museum visitor network, the Facebook forum network, the non-US airport route network, and the US 500 busiest commercial airports network. However, because the value of the edge weight *k* must be calculated for the WKPN algorithm in the weighted network, the time complexity was somewhat high. Furthermore, this study was aimed at the weighted-undirected networks; however, weighted-directed networks also exist widely in the real world. In further research, the WKPN algorithm will be improved for directed networks so as to be applicable to a wider range of fields. 

## Figures and Tables

**Table 1:** The node importance sorting results of the network, as shown in Figure 1.

Node No.	Weighted *K*-Order Propagation Number (WKPN) Algorithm		Mutual Information (MI) Algorithm	
	Node Importance	Sort	Node Importance	Sort
1	3.60	5	−1.96	5
2	2.32	7	−2.77	7
3	6.18	3	5.30	1
4	6.78	1	2.19	3
5	0	9	−2.77	7
6	0	9	−2.77	7
7	6.78	1	2.19	3
8	6.18	3	5.30	1
9	3.60	5	−1.96	5
10	2.32	7	−2.77	7

**Table 2:** Average efficiency of the network in Figure 1 before and after the corresponding node is deleted.

Network Characteristic	Initial Network	Deleting the Most Important Node, $v_{3}$ or $v_{8}$, in the MI Algorithm	Deleting the Most Important Node, $v_{4}$ or $v_{7}$ in the WKPN Algorithm
Average Efficiency *e*	0.2931	0.2529	0.2084
Decline of Average Efficiency *e*	0	0.0402	0.0847
Decline Rate ε	0	13.72%	28.90%

**Table 3:** Basic features of the Science Museum visitor network, Facebook forum network, the non-US airport routing network, and the US 500 busiest commercial airports network, including the number of nodes N, the number of edges E, and a short description.

Name of the Network	N	E	Description
Science Museum visitor	206	714	Weight stating the number of face-to-face contacts between visitors in the Science Museum.
Facebook forum	899	71,380	Nodes representing the forum users and the information communication between users and the weights of the edges indicating the number of pieces of information that have ever been sent.
Non-US airport routing	7976	15,250	Demonstrating the routing structure between two non-US airports.
US 500 busiest commercial airports	500	2980	Describing the structure of passengers traveling between the 500 busiest commercial airports.
